# Joy in the margins: examining narratives of everyday resistance among SGM BIPOC young adults in Orange County

**DOI:** 10.1186/s12889-025-25328-x

**Published:** 2025-12-30

**Authors:** Lindsay Asbury, Arianna Contestable, Tracy Tran, Chadi Maalouly, Sean Arayasirikul

**Affiliations:** 1 Department of Health, Society & Behavior, Joe C. Wen School of Population & Public Health, University of California, Irvine, Irvine, CA USA; 2 The Legacy Center, Joe C. Wen School of Population & Public Health, University of California, Irviine, Irvine, United States

**Keywords:** Joy, Sexual and Gender Minorities (SGM), Black, Indigenous And people of color (BIPOC), Resistance, Photovoice, Everyday resistance

## Abstract

**Background:**

Joy is a powerful and necessary counterpoint to the challenges faced by sexual and gender minoritized (SGM) communities. This study explores how SGM young adults of color in Orange County, California, cultivate joy as a form of everyday resistance and well-being in the face of systemic oppression.

**Methods:**

Drawing on a participatory action research approach, we used PhotoVoice to engage 19 Black, Indigenous, and people of color (BIPOC) SGM young adults as co-researchers. Participants documented and reflected on everyday experiences of joy through photographs and accompanying narratives, which were thematically analyzed.

**Results:**

Informed by Johansson and Vinthagen’s Everyday Resistance framework, we found that participants engaged in acts of detachment as a strategy for cultivating joy and resisting the demands of neoliberal productivity culture, including expectations of constant self-optimization, emotional endurance, and conformity.

**Conclusions:**

The study affirms the value of PhotoVoice in capturing the complex interplay between identity, environments, and systemic oppression. Centering joy in public health research can inform more affirming and culturally responsive interventions.

**Supplementary Information:**

The online version contains supplementary material available at 10.1186/s12889-025-25328-x.

## Introduction

Joy is a powerful and necessary counterpoint to the challenges faced by sexual and gender minoritized (SGM) communities. Scholars argue that research centered on joy disrupts dominant narratives that portray queer existence as inherently problematic by presenting more holistic and affirming perspectives on the lives of SGM individuals [28, 43, 53]. However, scientific literature reveals that joy remains underexamined in mainstream academic discourse, especially in the context of Black, indigenous, and people of color (BIPOC) SGM lives. Explorations of the topic have focused on joy in the context of resilience [17], the relationship between happiness and varying SGM identities [50], differing physical and mental health indicators among age-based cohorts [39], and the psychological components of joy-making [14]. While these studies demonstrate that joy serves as a form of resistance and affirmation, the scope of the work does not fully capture the everyday strategies that BIPOC SGM communities employ to cultivate joy in response to structural oppression.

In this study, we approach joy as an affective state reflecting a “distinct positive emotion” [55]. This conceptualization is particularly relevant given the disproportionate rates of adverse mental health outcomes and lower life satisfaction experienced by SGM communities, which are often linked to broader social and economic inequalities such as discrimination, stigmatization, limited access to healthcare, and inadequate financial resources [15, 24, 31, 50]. Douglass et al. [15] applied the minority stress model to examine life meaning among SGM individuals and found that anticipated rejection and identity concealment were negatively associated with individuals’ sense of meaning in life. Hajo et al. [24] found that positive mental health was less prevalent among SGM individuals, with most reporting lower life satisfaction compared to their heterosexual and cisgender counterparts. Focusing on trans and nonbinary adults in the United States, Kaufman et al. [31] identified social factors influencing life satisfaction and negative affect, reporting less satisfaction and more negative emotions among trans and nonbinary individuals in comparison to their cisgender counterparts. Thomeer and Reczek [50] broadened their analysis to examine social and economic influences, which revealed a clear association between structural inequities and lower levels of happiness among sexual minoritized groups. Compounding these challenges, studies have found that some racially and ethnically minoritized groups experience higher rates of homophobic victimization and discrimination within their own communities, which can further contribute to psychological distress [6, 48].

Amid the intersecting social and structural challenges facing BIPOC and SGM communities, much of the current research still frames joy as an individual mental health outcome, often disconnected from the lived experience of navigating systemic adversity. For example, Thomeer and Reczek [50] examined how sexual identity and behavior over the life course related to self-reported happiness, finding that identifying as bisexual, gay, or lesbian, or having both-sex partners, was often associated with lower happiness levels compared to those who identified as heterosexual. Russell et al. [39] analyzed health and well-being indicators among sexual minoritized individuals across age cohorts and found that younger cohorts reported poorer psychological health compared to their older counterparts. While these studies highlight significant disparities in mental health and well-being across identity categories and age groups, they provide limited insight into the lived experiences, relational contexts, and everyday practices through which SGM individuals cultivate joy in the face of systemic adversity.

Emerging research has begun to address these gaps by examining the process of joy-making. Edwards et al. [17] used open-ended survey responses from a diverse participant pool to consider how SGM individuals describe the sources of joy and pride in their lives. Drawing on these responses, they developed a framework of resilience-promoting resources. However, their study was limited by the nature of the data, which lacked the depth and contextual richness typically offered by more interactive or dialogic qualitative methods. Meanwhile, Denis et al. [14] focused on Black queer joy, using an online survey of 257 people and a theoretical framework to examine its origins and meanings. They highlighted the centrality of social connection, self-celebration, and other mechanisms for accessing joy and making sense of intersectional experiences. Their study emphasizes the value of grounding research on joy in the lived experiences of Black queer individuals, expanding how the concept is understood and applied.

Yet to fully understand the role of joy in the lives of BIPOC and SGM individuals, it is essential to situate these findings within the broader structural and sociopolitical contexts in which they are embedded. Issues faced by BIPOC and SGM individuals are both rooted in and exacerbated by conservative agendas in the United States that promote harmful rhetoric and policies, such as racist anti-immigration laws, anti-trans legislation and erasure, attacks on bodily autonomy, barriers to reproductive health care, and efforts to dismantle diversity, equity, and inclusion initiatives. Joy-based research highlights how SGM individuals experience meaning, connection, and happiness, rather than deficit-based approaches that focus on adverse experiences. As queer theorist Sara Ahmed poignantly notes, joy in the context of systemic oppression is a necessary and accessible form of resistance, validating the rights of SGM people to exist and thrive:

“...pleasures can allow bodies to take up more space… Indeed, the publicness of pleasure can function as a form of aggression; as a declaration of ‘We are here.’...Pleasure involves not only the capacity to enter into, or inhabit with ease, social space, but also functions as a form of entitlement and belonging” [3].

### Centering context and lived experience using photovoice

Building on this literature and motivated by the sociopolitical climate, the present study investigates how SGM individuals from diverse racial and ethnic backgrounds experience and cultivate joy in their everyday lives in response to structural oppression. Conducted in politically conservative-leaning Orange County, California, our research focused on BIPOC SGM young adults who face distinct challenges, particularly in light of a recent surge in anti-trans and anti-queer legislation. Several recent local events illustrate this growing hostility. In 2023, the Orange County Board of Supervisors implemented a law prohibiting the display of non-government flags on county property, signaling a response to the previous administration’s decision to fly LGBTQ + flags at county government sites. A similar law was enacted in Huntington Beach in 2024, when city officials banned the display of non-government flags on city property. Then, in early 2025, they installed a plaque featuring MAGA-related messaging at a public library. These symbolic actions sent a clear message of exclusion and reinforced discrimination against SGM community members, reflecting the broader anti-trans, anti-queer climate in the region.

Given the distinct challenges facing BIPOC SGM young adults in Orange County, we used PhotoVoice to explore how participants cultivate joy in their everyday lives. As a participatory action research (PAR) method that engages community members as co-researchers, PhotoVoice was well suited to our goals of centering marginalized voices and creating space for shared authority and collaboration [54]. Community-engaged research in suburban spaces, such as Orange County, positions participants as knowledge producers and collaborators in the interpretive process, shifting traditional power dynamics in research and creating space for participant agency in shaping findings [9]. Our research explores the mechanisms of joy by examining what enables it, the conditions under which it emerges, and the strategies people use to cultivate or pursue it. We investigated the contexts within participant lived experiences of joy and considered the personal and social factors vital to creating these experiences. This research is valuable to public health because it moves beyond abstract notions of resilience or individual mental health outcomes to identify structural conditions and culturally grounded strategies that support positive well-being, which provide insights for developing relevant and affirming recommendations and interventions. Approached through this lens, joy is not just an outcome,it functions as a protective factor against discrimination and supports long-term mental health.

### Everyday resistance

To better understand how BIPOC SGM young adults navigate structural oppression while cultivating joy, we drew on Everyday Resistance, a theoretical framework that explains how individuals and groups respond to domination through subtle, often hidden acts that challenge power without overt confrontation. The concept builds on James Scott’s foundational work, *Weapons of the Weak* [42], which describes resistance as a “mirror image” of domination, often enacted through small acts such as foot-dragging or evasion (p. 37). Building on and extending this work, Johansson and Vinthagen [30] reconceptualize everyday resistance through both materialist and poststructural perspectives, emphasizing that resistance is not always directly oppositional but instead emerges within diffuse, relational, and context-specific power dynamics. They identify four key dimensions: repertoires, or culturally embedded practices that may not seem political on the surface,relationships between actors, targets, and observers; and the spatial and temporal conditions that shape when and where resistance occurs. As Johansson and Vinthagen explain, “everyday resistance is manifested in various ways, and…it interacts dynamically in relation to historical social change and contextual differences" (2016, p. 432).

An everyday resistance framework provides tools for identifying who is resisting, what or whom they are resisting, and the spatial and temporal conditions under which resistance occurs. Scholars have used this framework to examine resistance to oppression [41], reframe resilience as a political act [47], and challenge the reproduction of stigma in everyday life [19]. For example, Frederick [19] draws on the concept of everyday resistance to examine how mothers with physical and sensory disabilities push back against intersecting forms of stigma rooted in cultural beliefs about disability, gender, and motherhood. By mapping resistance across multiple sites, such as the family, medical institutions, and schools, Frederick demonstrates how resistance is embedded in daily practices that take shape within unequal power relations. She shows how disabled mothers employ strategies like visibility politics, respectability politics, and disengagement to manage stigma. These practices, often unrecognized by others, illustrate the value of an Everyday Resistance framework in identifying the subtle and situated ways individuals contest power in ordinary settings.

In this study, we center our analysis on two key dimensions of Johansson and Vinthagen’s framework: repertoires of resistance and the spatial and temporal conditions that shape them (2016). Spatial repertoires emphasize that all acts of resistance are situated within specific social and material spaces. Resistance takes shape in discrete sites, such as homes, streets, or clinics, and is also shaped by broader spatial structures that regulate access, visibility, and mobility. Within these spatial repertoires, the body emerges as a key terrain of resistance, where power is inscribed and contested through everyday practices. In particular, marginal or “third” spaces offer alternative social imaginaries and relational geographies, where dominant norms can be disrupted and new forms of agency enacted. In addition to and alongside spatiality, Johansson and Vinthagen emphasize the temporal dimension of everyday resistance, arguing that resistance is also practiced in and through time as a central social condition. Just as space is socially constructed and regulated, so too is time, especially under neoliberal systems that value efficiency, productivity, and linear progression.

Neoliberal productivity is a system that organizes social and economic life through market-driven logics emphasizing personal responsibility, meritocracy, and the privatization of public goods. While it is related to broader systems of structural oppression, neoliberal productivity pressures and structural oppression are distinct: neoliberalism functions as a specific system of governance and economic logic that emphasizes self-responsibilization and market-based values, whereas structural oppression encompasses broader, historically rooted systems such as racism, heteronormativity, and patriarchy. These systems intersect and reinforce one another but operate through different mechanisms and scales. The demands of neoliberal productivity may appear to reward hard work and equal participation, but in practice, they obscure and sustain ongoing forms of racial and sexual exclusion. As Roberts [13, 38] show, BIPOC individuals are expected to succeed within a so-called “level playing field” that denies the reality of racism, attributing failure to personal flaws rather than structural inequality. For SGM people, especially those who disrupt heteronormative expectations of family and futurity, neoliberal systems mark them as unproductive or even threatening to social order, reinforcing their exclusion from full social and economic belonging [2, 16]. As a result, neoliberal productivity places uniquely intense pressures on BIPOC SGM individuals to conform to normative standards of value.

Temporal resistance, then, involves disrupting dominant time structures by reclaiming time for rest, slowness, or alternative rhythms of life that challenge normative expectations. By examining these repertoires, we highlight the practices that quietly but intentionally push back against neoliberal productivity norms, and the conditions in which they unfold to illuminate how resistance is both shaped by and responsive to lived spatial and temporal constraints.

## Methods

### PhotoVoice

This study employed qualitative methods, which prioritize attention to the detailed particulars of people’s understandings, interactions, and lived experiences to generate meaningful insights [44]. Unlike quantitative methodologies that are concerned with measurement and positivist models, qualitative approaches seek to understand the depth, complexity, and contextual specificity of social phenomena [44]. Central to these approaches is the recognition that knowledge is co-constructed through interactions between researchers and participants [18]. In this study, PhotoVoice and focus group discussions were used as complementary qualitative methods to illuminate how SGM young adults experience and create joy in their everyday lives.

PhotoVoice is a community-engaged participatory research methodology that aims to empower participants and their communities, encourage discussion of issues faced by a community, and facilitate social change [54]. PhotoVoice participants take photographs reflecting their experiences, share the meanings of their photos, and discuss the prominent patterns or themes captured [54]. Beyond its role as a tool for empowerment, PhotoVoice has also been used to examine the complex, relational, and embodied dimensions of young people’s lives [11] and to critically engage with the theoretical, methodological, and ethical assumptions underpinning participatory visual research in Indigenous contexts [27]. PhotoVoice is an accessible and feasible method for SGM communities and young adults, as it accommodates diverse forms of expression and creates space for stories that might otherwise remain unheard, particularly those shaped by exclusion or marginalization [21, 23, 32, 33, 52]. Participants attended a series of four study visits over Zoom. In the first study visit, participants were introduced to PhotoVoice, the purpose of the study, basic photography skills, and ethical practices for photographs. They took photos to address the following research question: “What does joy look like in your everyday life?” Before the next study visit, participants submitted two photos to research staff with written narratives explaining the meaning of the photos. During the follow-up study visit, each participant discussed their photos and the meaning behind them. After each individual’s share, other participants were invited to make meaning from their photo. Once everyone had shared their photos, all participants reflected on the collection of photos together. We elicited discussion to examine the meaning of photographs using a structured set of questions called SHOWeD questions [26, 32, 49], see Supplementary File 1). SHOWeD questions asked participants to respond to the following questions: What do you See here? What is really Happening? How does this relate to Our lives? Why does this situation, concern, or strength Exist? What can we Do about it? [26, 32, 49].

### Participants, recruitment, and screening

Eligible participants were between the ages 18 and 25 years old, identified as any racial or ethnic minoritized identity, identified as part of the LGBTQ + community, were able to read and speak English, and were a student at UCI or a resident of Orange County, California. All participants had a smartphone with the ability to take photos. Compensation for participation in this study was $200 provided at the completion of the four group sessions. Participants were recruited using social media, physical and electronic flyers, in-service presentations, and peer referral. Digital and physical recruitment flyers directed potential participants to email the research team. Following a phone screening to determine eligibility and collect demographic information, participants provided oral informed consent, completed a photo release form, and were provided a student information sheet. Twenty-three potential participants completed a phone screening for eligibility conducted by a research assistant, and 20 individuals were eligible to participate. Nineteen participants completed the study. This study and its procedures were reviewed and approved by the University of California, Irvine (UCI) Institutional Review Board (IRB #3874).

### Thematic analysis

Thematic analysis (TA) was utilized to identify prevalent themes for data analysis [7]. TA is a flexible and widely used method for analyzing qualitative data, particularly well-suited to identifying and interpreting patterns of meaning across participant narratives [7]. Reflexive thematic analysis is a more specific iteration of TA, which highlights the researcher’s positionality within the analytical process. Because RTA allows the researcher to conclude implied meanings in the data, the positionality of the researcher drawing the conclusions is important. Braun and Clarke [8] explain that even in inductive approaches to thematic analysis, it is impossible to entirely separate the researcher’s theoretical lens or positionality from the analytic process. Reflexive thematic analysis (RTA) acknowledges this subjectivity and rejects the idea of a single, objectively “correct” interpretation of the data (Byrne, 2021, p. 1393). Reflexive thematic analysis allowed the research team to critically reflect on their own perspectives and how these shaped the interpretation of participants’ narratives, while still enabling a systematic analysis of the data.

The data for this manuscript include participant photos, their written narratives, staff recorded field notes, and transcriptions of discussions. Data were uploaded to Dedoose for coding [40]. The research team (n = 3) employed RTA methods to examine recurring themes within participant statements [7]. We became familiar with the data by first reviewing all data and then generating codes inductively. Codes were applied to all textual data, reviewed, and discussed among the research team to generate emerging themes. These themes were then consolidated, separated, or modified based on conceptual overlap and relevance to the research question. Finally, a refined set of themes and subthemes was defined.

## Results

Twenty individuals aged 18 to 25 consented to participate in the study. Participants were recruited using social media (*n* = 6), a campus LGBTQ + center (*n* = 6), peer referral (*n* = 5), and email outreach (*n* = 3). Of the 20 participants who provided informed consent, 19 completed the study. The majority of participants were Asian (*n* = 11), and many were of more than one race/ethnicity (*n* = 5). The remaining participants were Latine or Hispanic (*n* = 2) and Black or African American (*n* = 1). Most participants were cisgender women (*n* = 10). Fewer participants were transgender men (*n* = 3), genderqueer or nonbinary (*n* = 2), questioning (*n* = 2), a transgender woman (*n* = 1), and a cisgender man (*n* = 1). Regarding sexual orientation, participants were largely bisexual (*n* = 6), followed by asexual (*n* = 4), queer (*n* = 3), pansexual (*n* = 3), lesbian (*n* = 2), and heterosexual (*n* = 1).

PhotoVoice data revealed a central theme of detachment from neoliberal productivity norms as a form of everyday resistance, which emerged through two subthemes: (1) resisting internalized and social pressures through intentional detachment and (2) disrupting routine as a temporal resistance strategy.

### Detachment from neoliberal productivity norms as a form of everyday resistance

For BIPOC SGM young adults in Orange County, intentional physical, emotional, or mental detachment from neoliberal productivity norms-based obligations emerged as a necessary and often deliberate strategy for cultivating joy. Using Johansson and Vinthagen’s [30] framework, we interpret participants’ actions as comprising two distinct repertoires of everyday resistance organized around detachment: (1) resisting internalized and social pressures through intentional detachment, and (2) disrupting routine as a temporal resistance strategy. Detachment emerged repeatedly throughout participants’ reflections, underscoring its centrality as a mechanism for experiencing joy.

#### Resisting internalized and social pressures through intentional detachment

**Fig. 1:**
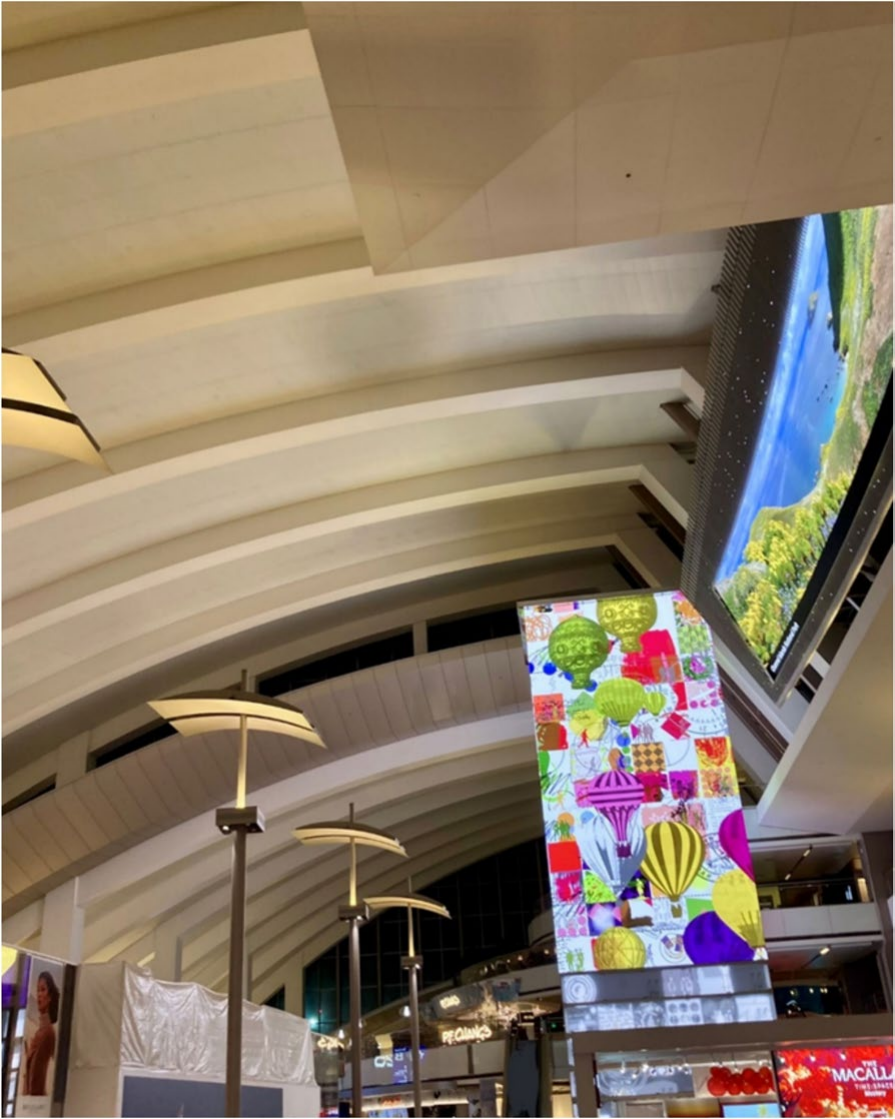
A quiet airport terminal captures the momentary relief of escape. The image reflects how physical distance offers temporary refuge from everyday pressures, allowing for detachment without the disruption of drastic change. It illustrates the tension between the urge to flee and the constraints of routine, where travel becomes a manageable act of resistance within systems that demand endurance

A recurring theme among young adults in this study was the perception of life as inherently chaotic and emotionally taxing. The stress associated with this broad sense of chaos was connected to a range of personal and societal challenges, spanning vague, unspecified concerns about the state of the world to clearly defined external expectations, such as the pressure to conform to idealized body standards or the cultural expectation of constant productivity. In this context, participants described intentional forms of detachment as a way to cope with overwhelming stress. Many described moments in which physical and symbolic spaces became sites of affirmation and resistance, which allowed them to step away from internal and social pressures and access forms of joy and authenticity. These spaces were experienced as radically different from the dominant environments participants typically navigated, which were often marked by surveillance, heteronormativity, and the pressures of neoliberal productivity. One participant described travel as a means of creating physical distance from daily stressors, illustrating how acts of detachment provided temporary relief from life's demands while also revealing the ongoing tension between the desire for escape and the pressure to return to routine:

“It can feel really good to put so much physical space between you and some of the day-to-day troubles of your life. …Travel helps me indulge that urge to run away from everything without actually making any drastic life changes, which is a feeling I often find myself looking forward to.” [Fig. 1]

The participant's response reflects a spatial repertoire of everyday resistance, where physical displacement becomes a strategy for coping without completely disrupting one’s daily life. The reference to avoiding “drastic life changes” underscores the constraints of routine and the perceived risks of noncompliance. In this view, detachment also requires managing one’s capacity within a system that demands endurance.

Another participant echoed this tension between obligation and pleasure, describing how constant demands from work and expectations of future success left little space for rest or joy:

“Sometimes I feel like there's just so much to do in my life, like work assignments, and then work on your future, …big corporations want people that really can give their 100% in the job…I think sometimes it's good to just go back to just what you like doing…you know, enjoying the little things.”

This reflection illustrates how small acts of enjoyment can function as a form of temporal resistance to the emotional toll of neoliberal productivity, interrupting the imperative of constant self-optimization.

Another participant described nature as a similarly reliable escape from daily stressors:

“...finding joy in nature…it's a way to just like detach from, I mean, your problems and like just the world like there's so many, you know, messed up things that go on, and I feel like nature is something that is just always like there for you to just experience.”

Here, detachment takes the form of immersion in the natural world, which is described as timeless, consistent, and emotionally grounding. This, too, represents a spatial and temporal repertoire of resistance, where the participant sought spaces that operate outside the fast-paced expectations of neoliberal productivity. In these settings, natural, undeveloped spaces can serve as physical and symbolic breaks from artificially produced environments. This finding echoes research that identifies nature and physical spaces as meaningful sources of joy and restoration for SGM individuals [14, 17].

**Fig. 2:**
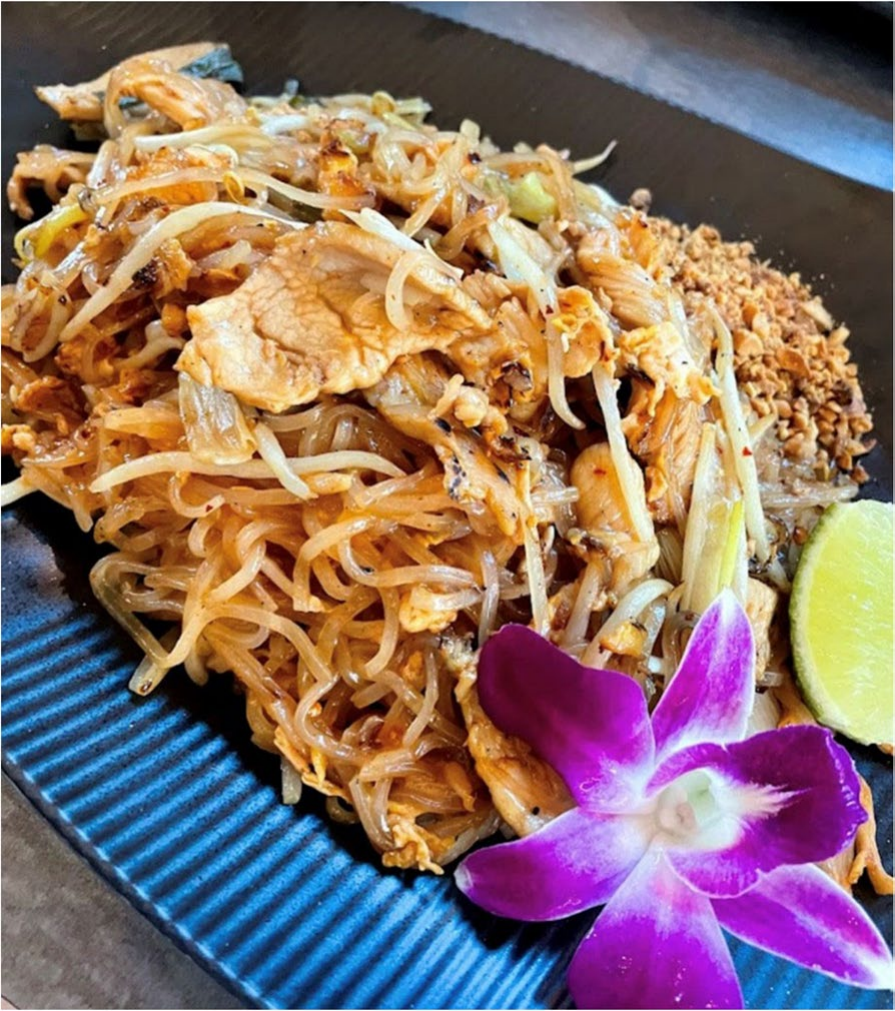
This image reflects an intentional act of reclaiming joy through food. In a culture that often attaches shame and restriction to eating, the participant frames this moment as a refusal of normative body expectations. Choosing to savor a favorite meal becomes an everyday act of resistance that centers comfort and sparks joy

Drawing on feminist and postcolonial theories of space, some locations can be understood as marginal or third spaces. These are zones of possibility forged at the edges of dominant culture ([5, 29], Soja 1996). For example, one participant described moments when resistance took the form of felt, visceral experiences that disrupted dominant norms of bodily discipline. Cultural pressures around ideal body standards were cited as barriers to experiencing joy, especially in moments meant to offer pleasure or comfort. The participant reflected on the challenge of overcoming internalized expectations around food and appearance:

“Eating food you love, not caring about anything beyond how it makes you feel is important to having a truly positive relationship with food. As someone who has struggled with restrictive eating, it is hard to internalize this message. Yet, when I eat something so good like Pad Thai at my favorite restaurant, I am able to see that food is meant to spark joy, not something that should make us feel bad.” [Fig. 2]

In this embodied form of spatial and temporal resistance, eating became a deliberate practice of reclaiming the body from restrictive norms. The participant prioritized present-moment pleasure over judgment or discipline to resist dominant expectations of bodily control, instead asserting food as a legitimate source of joy.

**Fig. 3:**
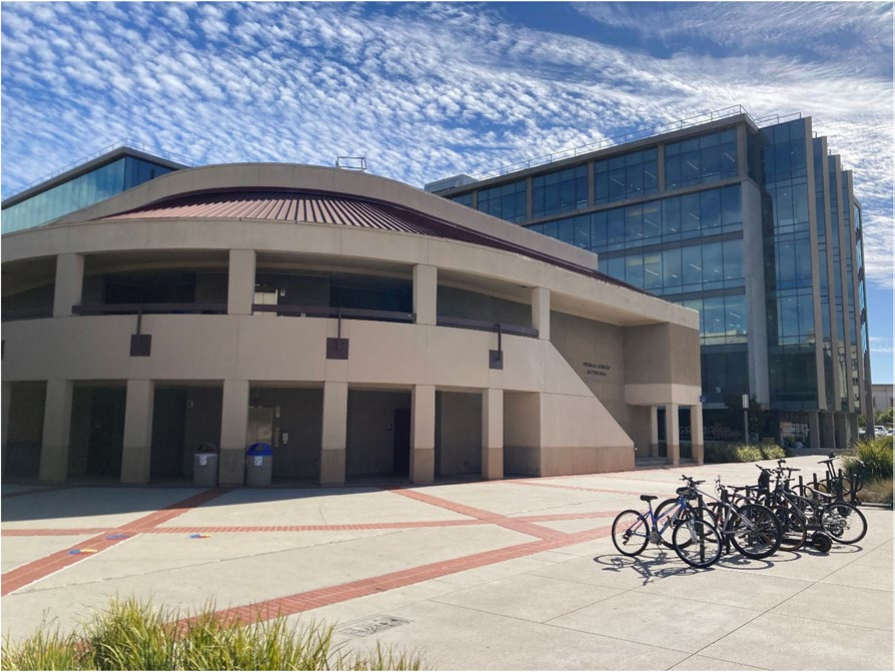
A participant shares a photo of the site where an anti-genocide divestment encampment once stood. Within this environment, everyday resistance helped create a temporary third space where normative structures of gender, power, and belonging were intentionally suspended. Here, queer visibility and self-expression were celebrated, offering a glimpse of a more liberated future, untethered from the constraints of heteronormative and institutional expectations

Another participant reflected on detachment from normative gender and sexuality scripts at a university protest encampment, describing it as a temporary community that restructured power and fostered queer visibility and belonging:

“Most of the rhetoric from University Admin pertaining to the encampment paints it as a violent and hostile space making people unsafe on campus. In truth, the encampment was a community space that attempted to liberate us from an oppressive and restrictive status quo. It was a safe space that was open and accepting to everyone united under its points of unity. In the encampment, everyone was equal, and existing structures of power and privilege were nearly abolished....my personal experience with the encampment was that it was absolutely a space of like joy and acceptance. It was where I finally—the first place that I felt comfortable introducing myself with my preferred pronouns. It was the first place on campus that I really felt comfortable living as my whole self.” [Fig. 3]

The creation of a spatial and embodied third space was a temporary yet transformative disruption to institutional norms that allowed for the expression of queer identity outside of hierarchies of gender, sexuality, and power. In this context, the participant’s queer self-presentation and use of preferred pronouns marked a refusal to comply with dominant expectations of a neoliberal heteronormative futurity in which bodies are guarantors of a reproductive and conforming social order [20].

#### Disrupting routine as a temporal resistance strategy

**Fig. 4:**
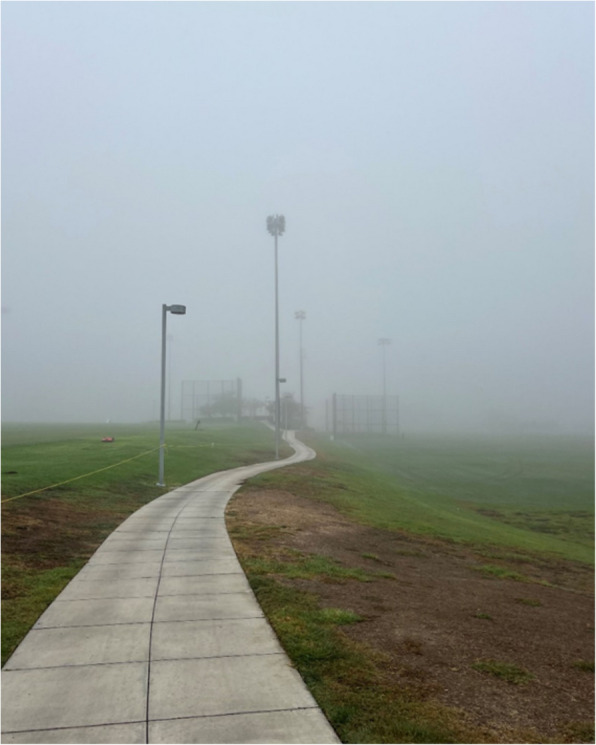
Even a subtle drop in temperature offers reprieve for one participant, who finds joy in the disruption of seasons and routines

The second subtheme explores the purposeful act of distancing from the monotony of daily life by deliberately disrupting familiar routines. Participants described engaging in new experiences, noticing seasonal changes, and briefly stepping outside their usual patterns as moments of renewal and opportunities for joy. These small acts created space to resist the forward-driving logic of constant productivity and introduced alternative relationships to time. Although participants did not explicitly identify neoliberalism as an agent of everyday resistance, their actions pushed back against its underlying pressures of performance and time optimization by reclaiming time for pleasure and presence. Engaging with something novel, like a new restaurant, made time outside the everyday routine feel more special and meaningful: “Going to this new place showed me how trying new things can bring joy. It’s a good reminder to step out of routine, explore new spots, and cherish the time with friends.” For one participant, even subtle changes, such as a drop in temperature, held symbolic value. These moments were experienced as opportunities to embrace variation and temporarily loosen the grip of structure:

“...I really like having routine in my life, but at the same time I also…really appreciate when there's like new things that happen. And [the cooler season] is just…representative of that, like it was like exciting to have the weather be different and not as sunny as it usually is…when I like walked outside and saw that it looks like foggy, it…made me happy… And it was like a nice change to my usual routine, while also still not being… crazy different to which I would like — I don't know — cause -like be inconvenient for me.” [Fig. 4]

**Fig. 5:**
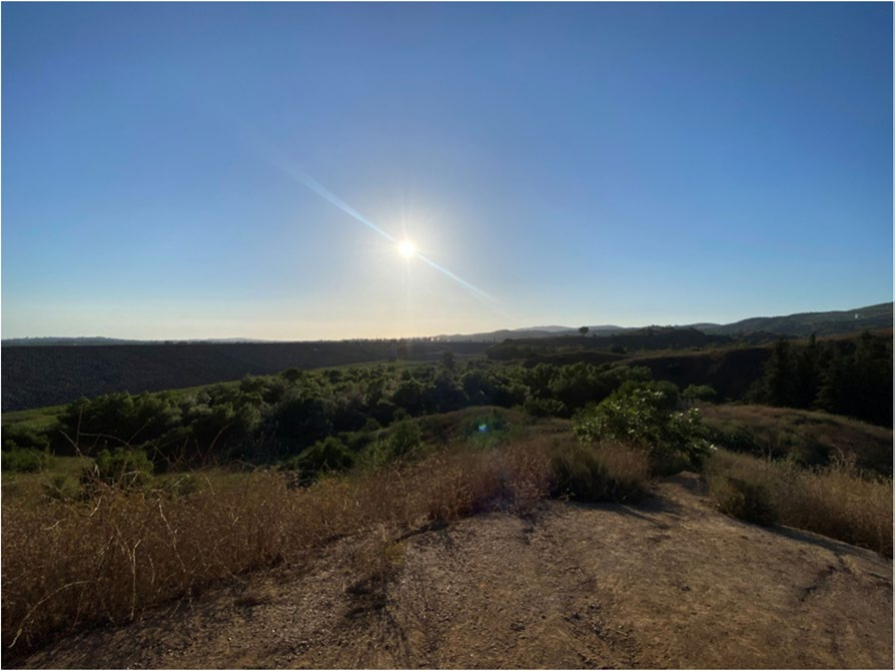
For one participant, hiking in nature represents a deliberate break from routine and a moment of pause that reclaims time from the demands of structure and productivity. The act reflects a subtle resistance to chrononormativity, carving out joy through intentional detachment. These pauses show how queer temporalities emerge by reshaping time as a resource for pleasure

These reflections illustrate a form of temporal resistance grounded in flexibility rather than rupture. Participants expressed a desire to resist monotony while still preserving the structure they relied on to manage responsibilities. This hesitation to fully detach reflects a broader tension, as moments of joy were often carved out within the boundaries of routine, not outside them. Rather than rejecting routine entirely, these moments reflect queer refusals of what Freeman describes as “chrononormativity,” a term that refers to the way social institutions organize time to encourage conformity, productivity, and normative life paths (2010, p. xxii). In contrast, participants enacted slow, affective temporalities that resisted productivity and linearity. Another participant highlighted this pattern, noting the importance of consistency while also naming the value of purposeful breaks as a way to shift how time was felt and used:

“I think that, while routines provide structure and routines can kind of reinforce dedication towards working towards goals, breaking that can kind of like stop the monotony of it all. Like, if we're just doing the same thing every single day, it kind of takes away from the time you get to invest in the things you love versus the things you have to do…To me, [hiking] kind of just symbolized that. It's easy to make it a part of your day when you take the time to invest in it, and it takes like intentionally and purposefully taking breaks…I like the act of physically breaking a routine to recharge somewhere else.” [Fig. 5]

In these examples, resistance emerged through intentional pauses and new rhythms that temporarily interrupted the time structures participants were expected to follow. These moments reflect the everyday reworking of time as a social and emotional resource that could be reclaimed to create meaning and pleasure. Together, these practices reflect what Halberstam [25] calls “queer time,” a temporal orientation grounded in the present and shaped by unscripted, non-normative possibilities. They also draw on Freeman’s [20] notion of “erotohistoriography,” which emphasizes how bodily pleasure and sensual experience can generate alternative ways of relating to history and time. In reclaiming these disruptions as meaningful, participants opened up possibilities for being that did not center on discipline or efficiency, revealing the subtle interplay between temporality, power, and everyday life, as well as a subtle, if unseen, resistance to neoliberal productivity norms.

## Discussion

This study used PhotoVoice to explore how BIPOC SGM young adults in Orange County create joy in ways that resist neoliberal, heteronormative expectations. Neoliberalism demands self-discipline, future orientation, and constant productivity, which are pressures that are compounded for those whose race, gender, or sexuality already position them outside the normative ideal. Against this backdrop of structural inequities, participants described joy as intentional, resistant, and relational. Their practices disrupted normative timelines and spatial expectations, offering insight into how detachment, spatial reconfiguration, and alternative temporalities can serve as everyday strategies of resistance to neoliberal productivity pressures, which compound existing forms of oppression they face. PhotoVoice has proven to be both a methodologically sound and conceptually aligned approach for exploring the abstract and complex experiences of SGM individuals. It has been effectively used to examine abstract and complex facets of experiences of SGM people, such as coming out [32], internalized transphobia [36], and queer creative practices [57], demonstrating its utility in capturing nuanced identity-related experiences. Its adaptability across diverse populations and settings is well-documented, as it has been employed to investigate and showcase the experiences of SGM young people [23, 32], trans women of color [52], and college students [21, 33]. Given its participatory and expressive nature, PhotoVoice was well-suited for the current study, which aimed to gather insight into the experiences of young people with marginalized identities. This topic is often overlooked or flattened in quantitative approaches. By engaging participants as co-researchers and grounding inquiry in their lived experiences, PhotoVoice facilitated meaningful reflection and community connection while supporting the broader goals of beneficial change and solutions for community members.

While several studies on SGM joy highlight resilience as a key theme, detachment has not been identified as a deliberate or proactive step to facilitate joy [14, 17]. Much of the existing research on joy focuses on the resilient act of recovery after harm, or bouncing back after experiencing adversity. While resilience is often celebrated, it has also been critiqued for placing the responsibility for managing structural oppression on individuals rather than systems [12]. Participants in this study often described detachment as an intentional strategy for navigating personal and external stressors. These responses parallel accounts of navigating difficult life experiences related to BIPOC and/or SGM identities found in existing scholarship [14, 17, 31, 50], particularly pertaining to SGM youth [39]. In contrast to narratives that frame joy as a response to harm, participants in this study described detachment as a proactive and intentional practice that involved stepping away from harmful systems rather than adapting to them. These moments of disengagement were framed as deliberate refusals to internalize the pressures of constant self-improvement and emotional endurance, thereby challenging the neoliberal logic that equates worth with productivity and resilience with individual responsibility. Our research shows how participants carved out space for rest and pleasure on their own terms, resisting the demand to be legible through hardship alone.

These findings resonate with and build upon existing scholarship that frames joy as a powerful strategy of resistance, particularly for BIPOC and queer communities. Across this literature, joy is understood as a deliberate and affirming response to oppression that asserts identity, fosters community, and reclaims agency in the face of structural marginalization [14, 37]. Participants in this study echoed these themes by intentionally stepping away from neoliberal expectations of productivity and discipline, engaging in practices that prioritized rest and pleasure. This aligns with frameworks that conceptualize joy as a tool for imagining more just and livable futures, particularly for queer people of color [51] and young people (Ginwright, 2016). Similarly, Youth Participatory Action Research demonstrates how creative, participatory approaches can foster collective agency and envision transformative possibilities for young people navigating systemic injustice [45, 56]. Even in digital spaces, joy has been shown to operate as cultural resistance,Lu and Steele [35], for example, highlight how expressions of Black joy online challenge dominant narratives and affirm cultural belonging. Similarly, participants in the present study used joy to disrupt normative scripts of time and space, asserting presence and relationality as forms of resistance against neoliberal systems that seek to regulate both. These findings affirm that when practiced intentionally by SGM BIPOC individuals, joy is a radical and embodied act of resistance and empowerment.

Our research showed that detachment served as a form of everyday resistance and a precursor to experiencing joy. Drawing on Johansson and Vinthagen’s [30] framework, we interpret deliberate detachment as a spatial and temporal repertoire that, though not always consciously framed as resistance by participants, functions in opposition to dominant power structures, creating the conditions of possibility for experiencing joy.

### Limitations

This study has limitations shaped by its context and methods. First, the findings may not reflect the experiences of BIPOC SGM young adults in regions outside of Orange County, California. Because participants opted into the study, there is potential for self-selection bias, as those who chose to participate may differ from those who did not. Additionally, we recognize that there are methodological limitations in our reliance on focus groups for PhotoVoice submission discussion. One-on-one semi-structured in-depth interviews may have allowed for more insight into the particularities of participant experiences with specific structurally oppressive factors. The reliance on self-reported narratives also introduces the possibility that responses were shaped by memory, social desirability, or the circumstances in which they were shared. Additionally, while PhotoVoice offered a powerful mode of expression, not all participants may have felt equally comfortable using photography to convey their emotions or experiences.

Although some participants may experience discomfort using photography to document and share their experiences, using PhotoVoice allowed flexibility and accessibility for exploring and discussing nuanced and emotionally complex topics. Participants were able to select images and craft their own narratives. This fostered agency in the storytelling process and enabled them to communicate their experiences in ways that felt authentic, meaningful, and personally resonant. This flexibility makes PhotoVoice especially valuable for studying identity, belonging, and well-being among historically marginalized populations. Although limitations exist, the method’s focus on participant voice and control allowed participants to explore joy, vulnerability, and resistance in ways that might not have emerged through more conventional approaches.

### Public health implications and community-based solutions

By centering joy in public health research, scholars create space for presence, power, and affirmation, presenting a more complex view that acknowledges identity-based struggle but also affirms the vibrant, meaningful lives of SGM individuals [53]. This research can equip stakeholders to recognize, support, and expand the conditions that make joy possible, particularly by identifying how joy is already being accessed, sought out, and experienced by SGM people.

Given that a central aim of PAR methods like PhotoVoice is to engage community members in change-making processes that enhance their own well-being and that of their communities, participants in this study were invited to offer suggestions for sustaining joy in their lives and in the lives of those around them. When asked, “What can we do about it?” most participants echoed the core theme identified in this study, underscoring the significance of purposeful detachment from stressors and routine to access joy. As one participant shared, “Life can be hectic and stressful so being able to find a soothing place to get away from all the noise can be very valuable.” Others highlighted the need to protect natural spaces that foster joy: “If we're appreciating the natural environment around us, we should also be committed to preserving that and also thinking about how we're interconnected with our environment.” These insights can guide SGM-supportive groups in designing activities and events that promote joy, including the creation of spaces where community members feel affirmed and connected. In medical settings, similar practices have been shown to support both physical and spiritual healing, while also fostering resistance among those who engage in them ([1], Bergbom et al., 2021). Safe public spaces, in particular, have been identified as important sites for cultivating joy and community well-being [1]. Lastly, scholarly work has only begun to examine how joy might influence health, but some early insights point to its potential to foster a “feeling of freedom” ([34], p. 7).

This research also highlights how individuals already engage in everyday practices that generate joy, empowering them to recognize and build upon their strengths. In this way, joy becomes a form of resistance and expression of a refusal to accept systemic oppression, which serves as a reminder that joy is not only possible but already embedded in the daily life of marginalized individuals.

## Conclusion

This research reveals a new understanding of the intentional adaptive behaviors, particularly detachment, that BIPOC SGM individuals employ to access joy in the face of persistent identity-related stressors. These acts function as coping strategies and as forms of everyday resistance that challenge normative demands around productivity and emotional endurance. Future research should continue to explore how BIPOC SGM young adults identify risk, engage in self-monitoring, and cultivate conditions that foster emotional well-being. These practices are responses to structural oppression that intentionally reclaim time, space, and the right to joy.

## Supplementary Information

Supplementary Material 1.

## Data Availability

The data that support the findings of this study are not openly available due to reasons of sensitivity and are available from the corresponding author upon reasonable request. Data are located in controlled access data storage at The University of California, Irvine.
