# Supplementary material for: Joy in the margins: examining narratives of everyday resistance among SGM BIPOC young adults in Orange County
Source: BMC Public Health. 2025 Dec 30;25:4377. doi: 10.1186/s12889-025-25328-x (PMC12754885; doi:10.1186/s12889-025-25328-x)
Supplement: Supplementary file 1 — Supplementary Material 1. [file 12889_2025_25328_MOESM1_ESM.docx]

**Supplementary File: Semi-structured PhotoVoice Group Questions**

***Main Research Question:*** What does joy look like in your everyday life?

***SHOWeD Questions (to facilitate PhotoVoice group conversation):***

- What do you **S**ee here?
- What is really **H**appening?
- How does this relate to **O**ur lives?
- **W**hy does this situation, concern, or strength **E**xist?
- What can we **D**o about it?
